# Why do horseflies need polarization vision for host detection? Polarization helps tabanid flies to select sunlit dark host animals from the dark patches of the visual environment

**DOI:** 10.1098/rsos.170735

**Published:** 2017-11-08

**Authors:** Gábor Horváth, Tamás Szörényi, Ádám Pereszlényi, Balázs Gerics, Ramón Hegedüs, András Barta, Susanne Åkesson

**Affiliations:** 1 Environmental Optics Laboratory, Department of Biological Physics, ELTE Eötvös Loránd University, Pázmány sétány 1, Budapest 1117, Hungary; 2 Department of Zoology, Hungarian Natural History Museum, Bird Collection, Ludovika tér 2-6, Budapest 1083, Hungary; 3 Department of Anatomy and Histology, University of Veterinary Medicine, István utca 2, Budapest 1078, Hungary; 4 Department of Cognitive Neurosciences, University of Tübingen, Auf der Morgenstelle 28, Tübingen 72071, Germany; 5 Estrato Research and Development Ltd., Mártonlak utca 13, Budapest 1121, Hungary; 6 Department of Biology, Centre for Animal Movement Research, Lund University, Ecology Building, Lund 223 62, Sweden

**Keywords:** horseflies, tabanids, polarization vision, parasite–host interaction, visual ecology, imaging polarimetry

## Abstract

Horseflies (Tabanidae) are polarotactic, being attracted to linearly polarized light when searching for water or host animals. Although it is well known that horseflies prefer sunlit dark and strongly polarizing hosts, the reason for this preference is unknown. According to our hypothesis, horseflies use their polarization sensitivity to look for targets with higher degrees of polarization in their optical environment, which as a result facilitates detection of sunlit dark host animals. In this work, we tested this hypothesis. Using imaging polarimetry, we measured the reflection–polarization patterns of a dark host model and a living black cow under various illumination conditions and with different vegetation backgrounds. We focused on the intensity and degree of polarization of light originating from dark patches of vegetation and the dark model/cow. We compared the chances of successful host selection based on either intensity or degree of polarization of the target and the combination of these two parameters. We show that the use of polarization information considerably increases the effectiveness of visual detection of dark host animals even in front of sunny–shady–patchy vegetation. Differentiation between a weakly polarizing, shady (dark) vegetation region and a sunlit, highly polarizing dark host animal increases the efficiency of host search by horseflies.

## 1. Introduction

Horseflies are polarotactic insects, that is, they are attracted to linearly polarized light [1]. Horsefly males and females detect water by means of the horizontal polarization of water-reflected light [2]. This sensorial capability has at least six functions in horseflies [1]: (i–iii) Horsefly males and females seek water to (i) drink, (ii) bath and (iii) mate with conspecifics near water. (iv–v) Horsefly females look for water (iv) to lay eggs onto water plants or into mud and (v) to rest on plants at the edge of water bodies in order to wait for host animals coming to drink and/or bath. (vi) In horsefly males and females, host finding is facilitated by polarotaxis elicited by the degree of polarization *d* of reflected light independently of the direction of polarization: females look for host animals to suck blood which is necessary to egg development, while males seek hosts to mate with the host-attracted females. Owing to these six vital functions, horsefly males and females have evolved to become strongly polarotactic insects.

Many haematophagous insects require a blood meal for egg production. For non-autogenous females, such blood is essential, while autogenous females can lay their first eggs without it. However, the blood meal increases egg production (e.g. [3–6]). Fecundity may further increase when blood is extracted from warm-blooded animals when compared with cold-blooded ones [7]. Owing to the required amount of blood sucked for egg laying, usually several hosts have to be visited by a female blood-sucking insect, thus more efficient host detection becomes an advantage [8–19]. As a consequence, biting insects may spread lethal pathogens to their hosts, including humans [11,20,21].

To take a blood meal, horsefly females need to recognize suitable hosts and differentiate them from the surrounding landscape. Several blood-sucking insect species are dependent on vision for flight control and host detection [11], including reflected linearly polarized light (e.g. [22–24]). Host-seeking horsefly females are more active in open sunny areas than in shaded or forested areas, furthermore, many tabanid species also have their peak of daily activity at midday when the sunlight is very high [25–27]. Horsefly females prefer sunlit, dark-coated, especially black host animals [22,28], and their visual host choice is partly governed by means of the degree of polarization of coat-reflected light [23,29]. The darker the host and the higher the degree of polarization of host-reflected light, the larger is its attractiveness to horsefly females [1]. In horseflies, water detection is governed by horizontal polarization, because water surfaces usually reflect horizontally polarized light [30], while in visual host choice the degree of polarization plays an important role, rather than the direction of polarization [23]. The reason for this latter phenomenon is that the body surface of host animals can reflect light with all possible directions (horizontal, tilted and vertical) of polarization [22,24,31].

The observed double polarotaxis in horseflies has been explored as the basis of polarization-based horsefly traps [32]: water-seeking horsefly males and females are attracted to these traps by horizontally polarized bait-reflected light, where the bait is a shiny (smooth) black horizontal surface (sticky board, oil surface or photovoltaic solar panel) mimicking a horizontally polarizing water surface [28,33,34]. Furthermore, host-seeking females are lured by vertical plane or spherical shiny (smooth) black targets suspended above the ground, imitating dark and strongly polarizing host animals [23,28,34]. Like polarotactic aquatic insects in general [35], horseflies are only weakly attracted to matt (rough) black or matt dark-coloured surfaces [2,22,23,36,37]. Therefore, the baits of most effective horsefly traps are black and smooth/shiny [32]. Smoothness is important, because only such surfaces can reflect light with high degrees of polarization near Brewster's angle *θ*_Brewster_ = arc tan(*n*), as measured from the normal vector of the reflecting surface, where *n* is the refractive index of the reflecting material.

Although horsefly females prefer sunlit dark and strongly polarizing host animals as well as shiny black targets in favour of matt, and thus weakly polarizing ones [22,23,36,37], the ecological and physical reasons for this are still unknown. To explain this behaviour, we formulate the following hypothesis: when a female horsefly is searching for a dark host, she selects dark patches as possible targets to take a blood meal. In the visual environment, however, there might be a mixture of dark patches in the surrounding, associated with vegetation (bushes and trees) which are not the preferred targets (dark host animals). Because leaves of trees and bushes do not have a particular orientation, they reflect light with all possible directions of polarization. The optical consequence of this is a low degree of polarization of dark vegetation patches as averaged by the ommatidia of the compound eyes of horseflies. On the other hand, the usually smooth (shiny) body surface of sunlit, dark host animals reflects light with high degrees of polarization. Thus, dark regions of the background vegetation and sunlit dark host animals may differ significantly in the degree of polarization of reflected light. Horseflies are polarization sensitive, thus they can distinguish a dark and weakly polarizing region of the vegetation from a sunlit dark and highly polarizing host animal.

In this work, we tested the above hypothesis. Using imaging polarimetry, we measured the reflection–polarization patterns of a dark host model and a black cow in front of different vegetation backgrounds and under various illumination conditions. We compared the intensities and degrees of polarization of light originating from dark patches of vegetation and the dark model/cow. We determined the chances of successful host-selection driven solely by intensity or degree of polarization or the combination of both. We show that information on the degree of polarization considerably increases the effectiveness of visual detection of sunlit dark host animals in front of vegetation backgrounds.

## 2. Material and methods

### 2.1. Imaging polarimetry

**Figure 1.**
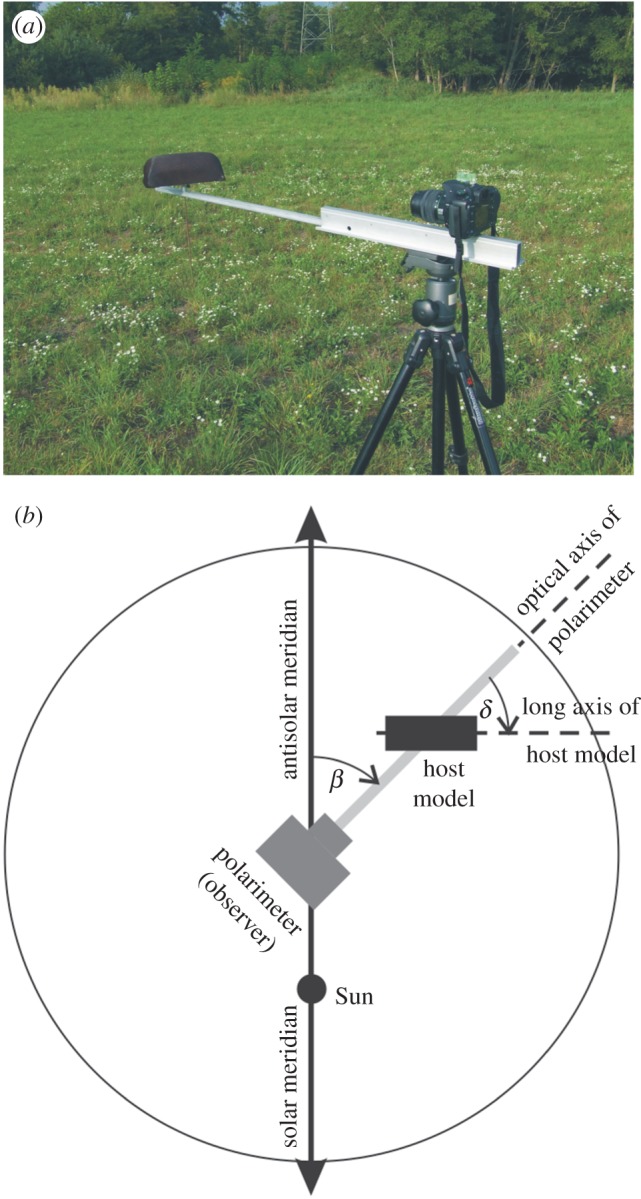
(*a*) Photograph of the set-up of our imaging polarimetric measurement with a host model (a half cylinder covered with a dark brown horse hide) in front of the polarimeter. (*b*) Geometry of the measuring arrangement seen from above. *β*: angle of the polarimeter's optical axis clockwise from the antisolar meridian, *δ*: angle of the long axis of the host model clockwise from the polarimeter's optical axis.

Using imaging polarimetry in the field, we measured the reflection–polarization patterns of a host model in front of various vegetation backgrounds (figure 1) in the red (650 nm), green (550 nm) and blue (450 nm) parts of the spectrum. The method of imaging polarimetry is described in detail elsewhere [38]. Measurements were performed in July 2016 at a Hungarian horse farm near the village Göd (47°43′ N, 19°09′ E) during sunny weather under a cloudless sky. The host model was a half cylinder (diameter = 10 cm, length = 22 cm) covered with a piece of dark bay (i.e. brown) horse hide (figure 1*a*), the hairs of which were oriented similarly as on the back and side of living horses. This host model imitated well the upper half of a semi-cylindrical host's body. The host model was fixed in front of the polarimeter at a distance of 1 m (figure 1). The horizontal orientation of the polarimeter's optical axis relative to the antisolar meridian (angle *β* in figure 1*b*) and the horizontal orientation of the model's long axis relative to the polarimeter's optical axis (angle *δ* in figure 1*b*) could be changed arbitrarily. We performed polarization measurements of this host model in front of different vegetation backgrounds composed of trees and bushes (figure 1*a*). The illumination characteristics of the scenes investigated, and the alignment angles *β* and *δ* of the polarimeter and the host model are given in table 1.

**Table 1.** Illumination characteristics of the nine different scenes investigated, and alignments *β* and *δ* of the polarimeter (observer) and the host model (figure 1*b*). *β*: angle of the polarimeter's optical axis clockwise from the antisolar meridian, *δ*: angle of the long axis of the host model clockwise from the polarimeter's optical axis.

scene	illumination characteristics	*β*	*δ*
1 (figure 3*a*)	sun is behind the polarimeter, host model is sunlit	0°	+30°
2 (figure 4*a*)	sun is behind the polarimeter, host model is sunlit	0°	+60°
3 (figure 5*a*)	sun is behind the polarimeter, host model is sunlit	0°	+90°
4 (figure 6*a*)	sun is behind the polarimeter, host model is sunlit	0°	−45°
5 (figure 7*a*)	sun is at the left, host model is shady	−90°	−45°
6 (figure 8*a*)	sun is at the left, host model is sunlit	−90°	−45°
7 (figure 9*a*)	sun is at the left, host model is sunlit	−45°	−45°
8 (figure 10*a*)	sun occluded by a cloud (at the left), cow is shady	−90°	−45°
9 (figure 11*a*)	sun is at the left, cow is sunlit	−90°	−45°

Imaging polarimetry of a living black cow was performed in September 2008 next to the Stensoffa Ecological Field Station (55°42′ N, 13°25′ E) near Lund, South Sweden. Owing to the partly cloudy sky, the cow was either sunlit or shady (when the sun was hidden by a cloud). We measured the reflection–polarization characteristics of this cow when she was motionless during suckling her black calf.

### 2.2. Host-detection algorithms

Using the patterns of the relative intensity *i* and degree of polarization *d* of reflected light measured in the red, green and blue spectral ranges for a given scene (with the host model and a vegetation background), the computer program (developed by us) first detected the pixels which were both sufficiently dark and polarized, that is where the following two conditions were satisfied: (i) 0% ≤ *i* ≤ i* and (ii) d* ≤ *d* ≤ 100% in the red (650 nm), green (550 nm) and blue (450 nm) spectral ranges, where *i* = 100*I*/*I*_max_ (*I* is the intensity, *I*_max_ = 255 is the maximum digital value of *I*), and i* and d* are threshold values. These detected pixels (*N*_detected_) of the scene could belong either to a host animal, or to the background. For a host-seeking polarization-sensitive horsefly, it is important that the majority of these detected points (*N*_detected_) should belong to the host animal. Finally, the software counted the number *N* of those detected pixels that were positioned on the host model. The ratio *r* = *N*/*N*_detected_ gives the recognition success, that is the efficiency of host detection, where *N*_detected_ is the number of all detected pixels. The larger the *r*, the higher is the chance of successful host detection. As a result, for a given scene we obtained the recognition success matrix *r*(i*, d*) as functions of the relative intensity threshold i* and the degree of polarization threshold d* (figure 3*h*,*i*). We distinguish three different host-detection algorithms:

**Algorithm 2.1**

The host is detected only on the basis of the relative intensity *i*, without taking into consideration the degree of polarization *d*. The results obtained with the use of this algorithm are in the horizontal row *r*(0% ≤ i* ≤ 100%, d* = 0%) of the recognition success matrix *r*(i*, d*).

**Algorithm 2.2**

The host is detected only on the basis of the degree of polarization *d*, without taking into consideration the relative intensity *i*. The results obtained with the use of this algorithm are in the vertical column *r*(i* = 100%, 0% ≤ d* ≤ 100%) of the recognition success matrix *r*(i*, d*).

**Algorithm 2.3**

The host is detected on the basis of both the relative intensity *i* and the degree of polarization *d*. The results obtained with the use of this algorithm cover the entire recognition success matrix *r*(0% ≤ i* ≤ 100%, 0% ≤ d* ≤ 100%), in general.

Note that, from a mathematical point of view, host detection algorithms 2.1 and 2.2 are special cases of algorithm 2.3, and thereby can be represented within the appropriate row and column of matrix *r*(i*, d*).

## 3. Results

Here we describe only scene 1 in detail, because we measured the same parameters and obtained quite similar conclusions for scenes 2–9 with a host model or a cow. Figure 3*a* shows the photograph of scene 1 containing the dark brown host model with a meadow and trees in the background when the sun was shining from behind the polarimeter (figure 3*c*). The target to be recognized by horseflies, i.e. the host model, is coloured red in figure 3*b*. According to the patterns of the degree of polarization *d* measured in the red, green and blue spectral ranges (figure 3*d*–*f*), the green vegetation is the most polarizing (reflects light with the highest *d*-values) in the blue, and is the least polarizing (reflecting light with the lowest *d*-values) in the green. The brown host model is also the most polarizing in the blue, while the least polarizing in the red.

**Figure 2.**
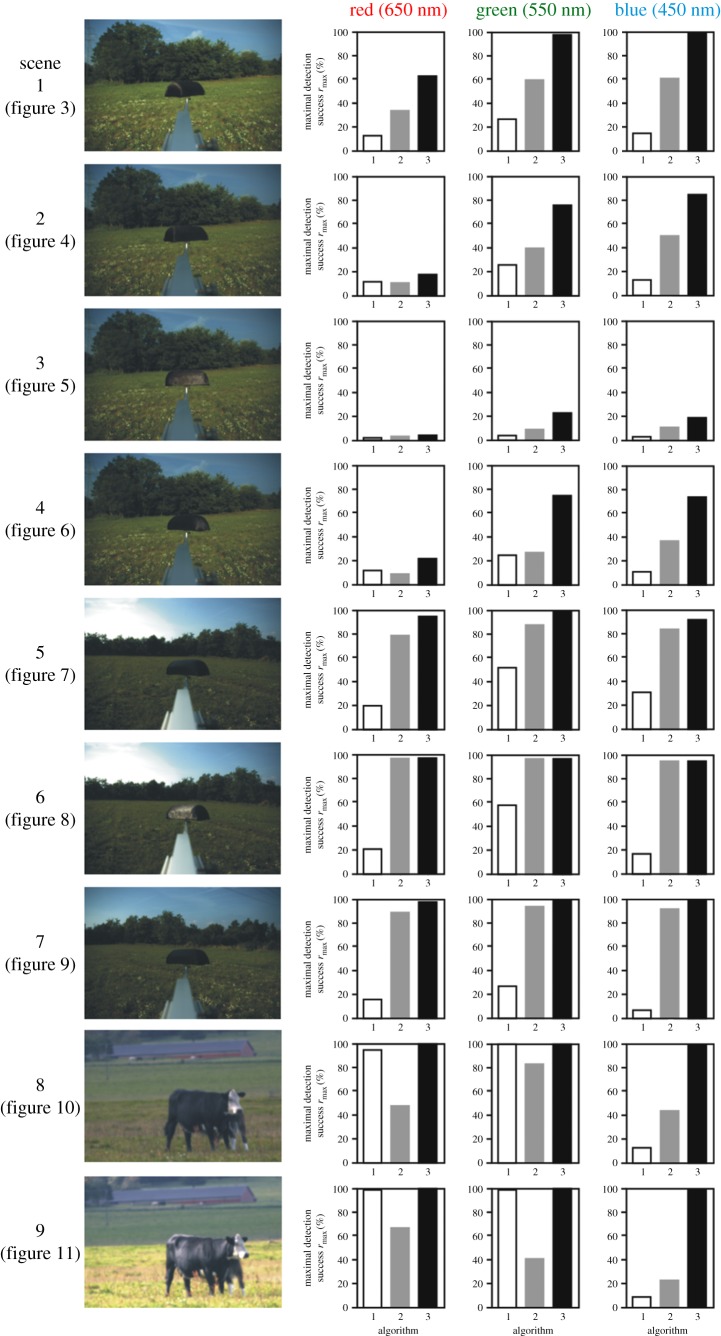
Maximum of the recognition success *r* (%) of the host model/animal obtained with the use of host detection algorithms 2.1 (white bars), 2.2 (grey bars) and 2.3 (black bars) in the red (650 nm), green (550 nm) and blue (450 nm) parts of the spectrum for the nine different scenes investigated. The numerical data visualized here are given in table 2.

**Figure 3.**
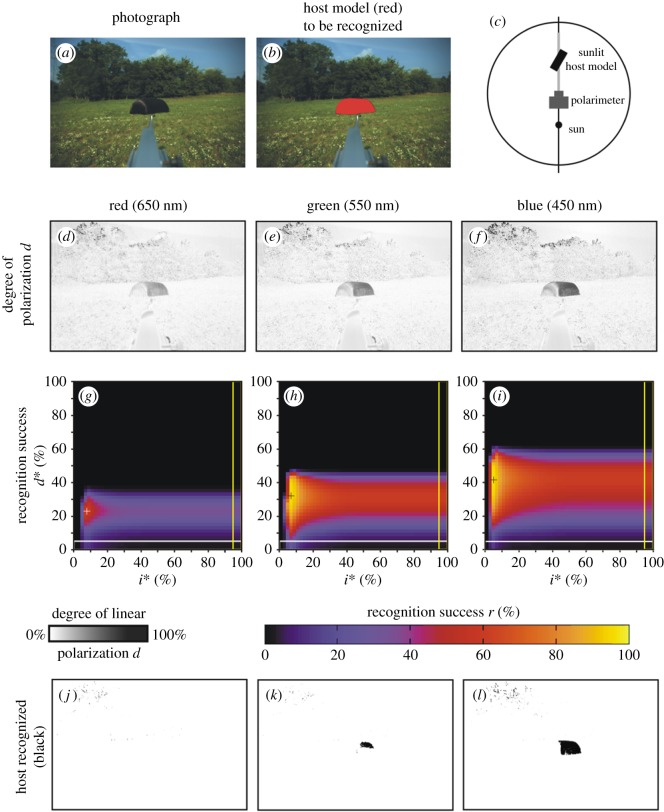
Scene 1. (*a*) Photograph of the dark brown host model with a meadow and trees in the background. (*b*) In this figure, the host model is artificially coloured red in order to display the target to be recognized by our algorithms (horsefly). (*c*) Geometry of the measuring arrangement. (*d*–*f*) Pattern of the degree of linear polarization *d* in the red, green and blue spectral ranges. (*g*–*i*) Recognition success *r* in the red, green and blue spectral ranges as functions of the thresholds d* of *d* and i* of the relative intensity *i* = *I*/*I*_max_, where *I*_max_ = 255 is the maximal intensity. The cross + shows the position of the maximum of *r* (table 2). The horizontal white-perimeter rectangle corresponds to the values d* = 0% and 0% ≤ i* ≤ 100%, when in host recognition polarization information is not taken into consideration, only intensity (table 2). The vertical yellow-perimeter rectangle corresponds to the values i* = 100% and 0% ≤ d* ≤ 100%, when in host recognition intensity information is not taken into consideration, only polarization (table 2). (*j*–*l*) Regions (black) of the scene recognized as dark and polarized host in the red, green and blue parts of the spectrum. Pixels are black for which the following conditions are satisfied: 0 ≤ *i* ≤ i* (dark enough) and d* ≤ *d* ≤ 100% (polarized enough), where i* = 4% and d* = 42% are the thresholds at which the recognition success *r* is maximal in the blue spectral range (shown in *l*).

Figure 3*g*–*i* shows the host recognition success *r*(i*, d*) in the red, green and blue spectral ranges as functions of the relative intensity threshold i* and degree of polarization threshold d* for scene 1. At a given i*, *r* depends strongly on d* and exhibits a steep maximum. On the other hand, at a given d*, *r* depends only slightly on i*, that is, *r* is nearly constant through a wide interval of i*. *r*(i*, d*) has one maximum (marked with symbol + in figure 3*g*–*i*), whose value and position in the i*–d* space are wavelength-dependent: $r_{\max}^{\mathrm{red}}$(i* = 8%, d* = 24%) = 63%, $r_{\max}^{\mathrm{green}}$(i* = 6%, d* = 32%) = 98%, $r_{\max}^{\mathrm{blue}}$(i* = 4%, d* = 42%) = 99% (table 2 and figure 2). Hence, the d*-value at which *r* is maximal increases with decreasing wavelength.

**Table 2.** Maximum of the recognition success *r*_max_(i*, d*) (%) of the host model/animal for relative intensity threshold i* (%) and degree of polarization threshold d* (%) (algorithm 2.3: when in host recognition both the degree of polarization *d* and intensity *i* are taken into consideration), the minimum–maximum interval *r*_min_ – *r*_max_ of recognition success *r* for d* = 0% and 0% ≤ i* ≤ 100% (algorithm 2.1: when in host recognition the degree of polarization *d* is not taken into consideration, only the intensity *i*), and the minimum–maximum interval *r*_min_ – *r*_max_ of *r* for i* = 100% and 0% ≤ d* ≤ 100% (algorithm 2.2: when in host recognition the intensity *i* is not taken into consideration, only the degree of polarization *d*) studied in the red (650 nm), green (550 nm) and blue (450 nm) parts of the spectrum for the nine different scenes investigated. These data are visualized in figure 2.

scene	recognition success *r* (%)	red (650 nm)	green (550 nm)	blue (450 nm)
1 (figure 3*a*)	*r*_min_–*r*_max_ (algorithm 2.1)	0–13	0–27	0–15
	*r*_min_–*r*_max_ (algorithm 2.2)	0–34	0–60	0–61
	*r*_max_(i*, d*) (algorithm 2.3)	63 (8, 24)	98 (6, 32)	99 (4, 42)
2 (figure 4*a*)	*r*_min_–*r*_max_ (algorithm 2.1)	0–12	0–26	0–13
	*r*_min_–*r*_max_ (algorithm 2.2)	0–11	0–40	0–50
	*r*_max_(i*, d*) (algorithm 2.3)	18 (12, 14)	76 (8, 22)	85 (6, 32)
3 (figure 5*a*)	*r*_min_–*r*_max_ (algorithm 2.1)	0–2	0–4	0–3
	*r*_min_–*r*_max_ (algorithm 2.2)	0–3	0–9	0–11
	*r*_max_(i*, d*) (algorithm 2.3)	4 (16, 12)	23 (6, 24)	19 (10, 30)
4 (figure 6*a*)	*r*_min_–*r*_max_ (algorithm 2.1)	0–12	0–25	0–11
	*r*_min_–*r*_max_ (algorithm 2.2)	0–9	0–27	0–37
	*r*_max_(i*, d*) (algorithm 2.3)	22 (8, 18)	75 (6, 26)	74 (4, 26)
5 (figure 7*a*)	*r*_min_–*r*_max_ (algorithm 2.1)	0–20	0–52	0–31
	*r*_min_–*r*_max_ (algorithm 2.2)	0–79	0–88	0–84
	*r*_max_(i*, d*) (algorithm 2.3)	95 (6, 36)	99 (6, 38)	92 (6, 44)
6 (figure 8*a*)	*r*_min_–*r*_max_ (algorithm 2.1)	0–21	0–58	0–17
	*r*_min_–*r*_max_ (algorithm 2.2)	0–97	0–97	2–95
	*r*_max_(i*, d*) (algorithm 2.3)	97 (78, 70)	97 (82, 72)	95 (68, 72)
7 (figure 9*a*)	*r*_min_–*r*_max_ (algorithm 2.1)	0–16	0–27	0–7
	*r*_min_–*r*_max_ (algorithm 2.2)	0–89	0–94	0–92
	*r*_max_(i*, d*) (algorithm 2.3)	98 (10, 48)	99 (8, 44)	99 (8, 56)
8 (figure 10*a*)	*r*_min_–*r*_max_ (algorithm 2.1)	0–95	0–100	0–13
	*r*_min_–*r*_max_ (algorithm 2.2)	0–48	2–83	2–44
	*r*_max_(i*, d*) (algorithm 2.3)	100 (10, 20)	100 (10, 2)	99 (20, 92)
9 (figure 11*a*)	*r*_min_–*r*_max_ (algorithm 2.1)	0–99	0–99	0–9
	*r*_min_–*r*_max_ (algorithm 2.2)	2–67	2–41	2–23
	*r*_max_(i*, d*) (algorithm 2.3)	100 (10, 4)	100 (14, 16)	99 (18, 56)

If horseflies detected dark hosts with algorithm 2.1 (using only *i*-values), their host recognition success *r* versus i* would be the lowermost white-perimeter horizontal row with d* = 0% in the matrices *r*(i*, d*) of figure 3*g*–*i*. According to table 2 and figure 2, in this case the ranges of *r* would be: 0% ≤ *r*_red_ ≤ 13%, 0% ≤ *r*_green_ ≤ 27%, 0% ≤ *r*_blue_ ≤ 15%. Should horseflies detect host with algorithm 2.2 (using only *d*-values), their host recognition success *r* versus d* would be the right yellow-perimeter column with i* = 100% in the matrices *r*(i*, d*) of figure 3*g*–*i*. Then, the resulting *r*-ranges are: 0% ≤ *r*_red_ ≤ 34%, 0% ≤ *r*_green_ ≤ 60%, 0% ≤ *r*_blue_ ≤ 61% (table 2 and figure 2). If dark hosts are detected with algorithm 2.3 (using both *i*- and *d*-values), the maxima of *r* (marked with + in figure 3*g*–*i*) are: $r_{\max}^{\mathrm{red}}$ = 63%, $r_{\max}^{\mathrm{green}}$ = 98%, $r_{\max}^{\mathrm{blue}}$ = 99% as we have seen above (table 2 and figure 2). Hence, the use of algorithm 2.3 results in 63/13 = 4.8-times, 98/27 = 3.6-times, 99/15 = 6.6-times (algorithm 2.3 versus 2.1) and 63/34 = 1.9-times, 98/60 = 1.6-times, 99/61 = 1.6-times (algorithm 2.3 versus 2.2) larger host recognition success *r* than using algorithm 2.1 or 2.2, in the red, green and blue spectral range, respectively. Furthermore, using only *d*-values (algorithm 2.2) results in 34/13 = 2.6-times, 60/27 = 2.2-times, 61/15 = 4.1-times larger host recognition success *r* than using only *i*-values (algorithm 2.1) in the red, green and blue spectral range, respectively. From this we conclude that the degree of polarization *d* helps horseflies to separate the dark host animals from the dark patches of their visual environment, and this is the reason why horseflies are more successful when using polarization vision in their host choice.

Figure 3*j*, *k* and *l* displays the regions recognized as host in the red, green and blue parts of the spectrum, respectively, for scene 1. Here pixels are shown black for which 0 ≤ *i* ≤ i* and d* ≤ *d* ≤ 100%, where i* = 4% and d* = 42% are the thresholds at which *r* is maximal in the blue spectral range (figure 3*i*). It is clearly seen from figure 3*j*–*l* that the region detected as host with the use of algorithm 2.3 is largest in the blue part of the spectrum, it is much smaller in the green, and in the red no part of the host model is recognized (corresponding to *r* = 0%). On the other hand, in all three spectral ranges a few small areas (with lower *i*- and higher *d*-values) of the trees are erroneously detected as host (false positives).

**Figure 4.**
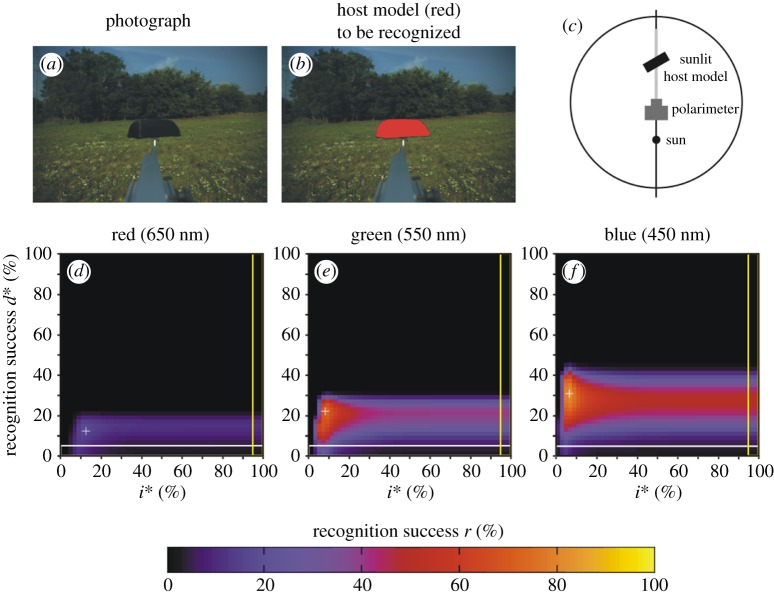
Scene 2. (*a*) Photograph of the brown host model with a meadow and trees in the background. (*b*) In this figure, the host model is artificially coloured red in order to display the target to be recognized by our algorithm (horsefly). (*c*) Geometry of the arrangement. (*d*–*f*) Recognition success *r* in the red, green and blue spectral ranges as functions of the thresholds d* of *d* and i* of the relative intensity *i* = *I*/*I*_max_, where *I*_max_ = 255 is the maximal intensity. Symbol (+) shows the position of maximum of *r* (table 2). The horizontal white-perimeter rectangle corresponds to the values d* = 0% and 0% ≤ i* ≤ 100%, when in host recognition polarization information is not taken into consideration, only intensity (table 2). The vertical yellow-perimeter rectangle corresponds to the values i* = 100% and 0% ≤ d* ≤ 100%, when in host recognition intensity information is not taken into consideration, only polarization (table 2).

**Figure 5.**
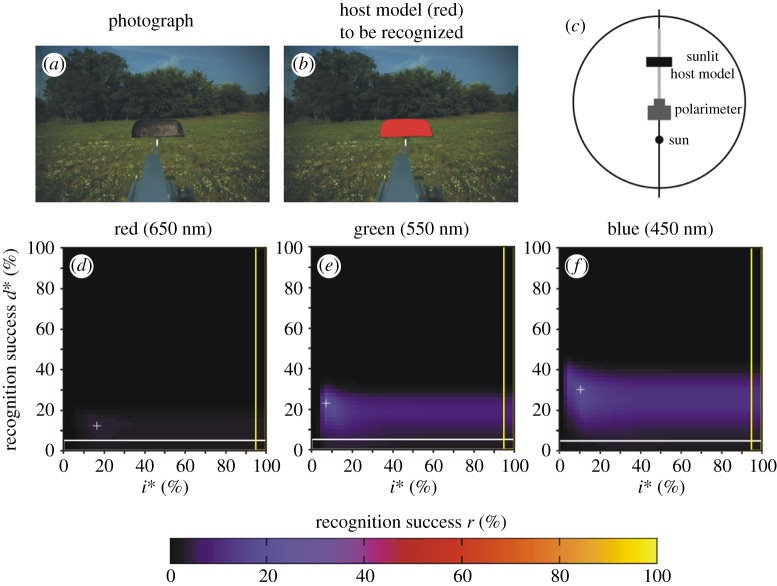
Scene 3. As figure 4 for the arrangement shown in panel (*c*) and for illumination conditions given in table 1.

**Figure 6.**
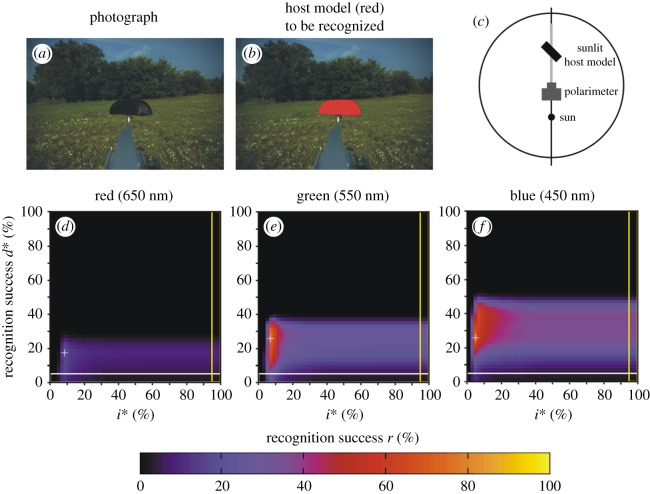
Scene 4. As figure 4 for the arrangement shown in panel (*c*) and for illumination conditions given in table 1.

**Figure 7.**
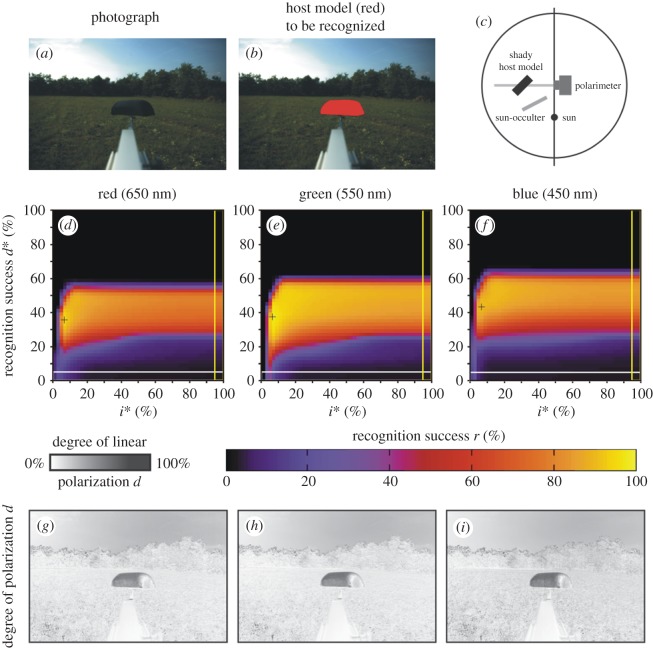
Scene 5. As figure 4 for the arrangement shown in panel (*c*) and for illumination conditions given in table 1. (*g*–*i*) Pattern of the degree of polarization *d* in the red, green and blue spectral ranges.

**Figure 8.**
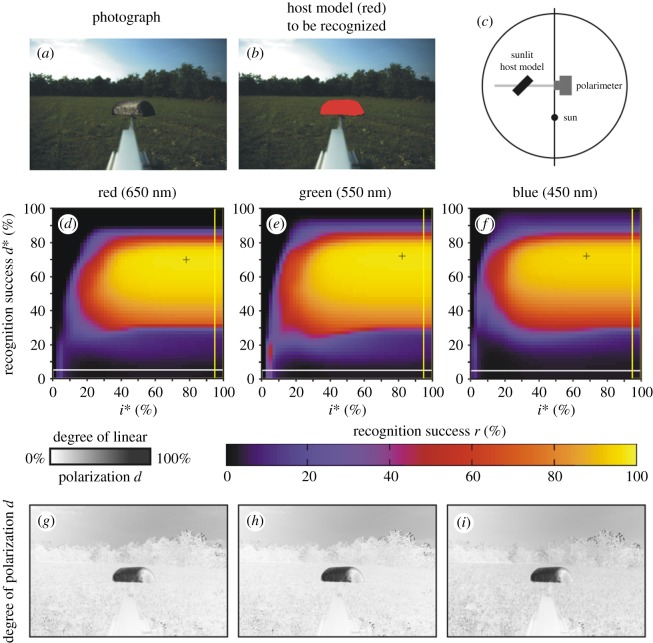
Scene 6. As figure 4 for the arrangement shown in panel (*c*) and for illumination conditions given in table 1. (*g*–*i*) Pattern of the degree of polarization *d* in the red, green and blue spectral ranges.

**Figure 9.**
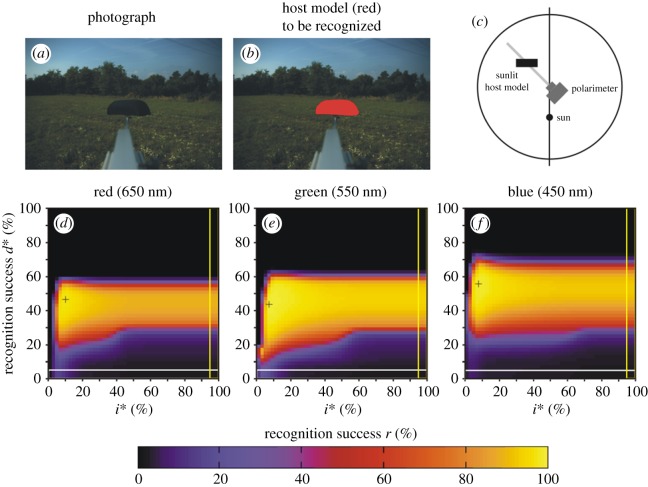
Scene 7. As figure 4 for the arrangement shown in panel (*c*) and for illumination conditions given in table 1.

The above conclusion is corroborated by the information presented in figures 2, 4–9 and table 2 obtained for six other scenes with different illumination conditions of the host model and various vegetation backgrounds. In all these investigated situations, in a given spectral range (red, green, blue) the maximum of *r* obtained with algorithm 2.3 is always much greater than that for algorithms 2.1 and 2.2 (figure 2 and table 2).

Figures 7 and 8 show scenes 5 and 6 where the host model has the same alignment angles *β* and *δ* in shady and sunlit situation, respectively. Figures 7*g*–*i* and 8*g*–*i* display the patterns of the degree of polarization *d* in the red, green and blue spectral ranges for scenes 5 and 6. We can see that in a given spectral range the sunlit host model reflects light with higher *d*-values than the shady model. The important consequence of this is the increase of the host recognition success *r*: from figures 2, 7*d*–*f*, 8*d*–*f* and table 2, it is clear that in a given spectral range the maximum of *r* obtained with algorithm 2.3 is larger when the host model is sunlit. Hence, polarization-sensitive horseflies can more easily detect sunlit host animals than shady ones, and this explains why they prefer sunlit hosts against shady ones.

**Figure 10.**
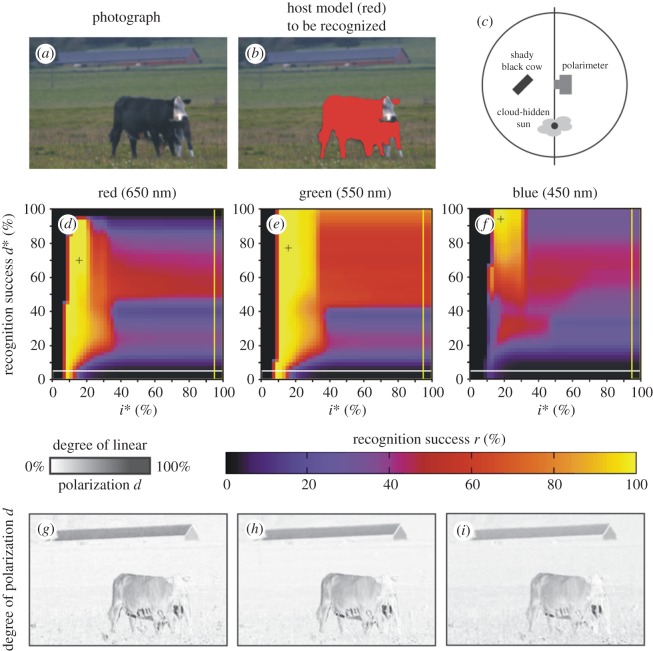
Scene 8. As figure 4 for a shady black cow. The arrangement is shown in panel (*c*) and the illumination conditions are given in table 1.

**Figure 11.**
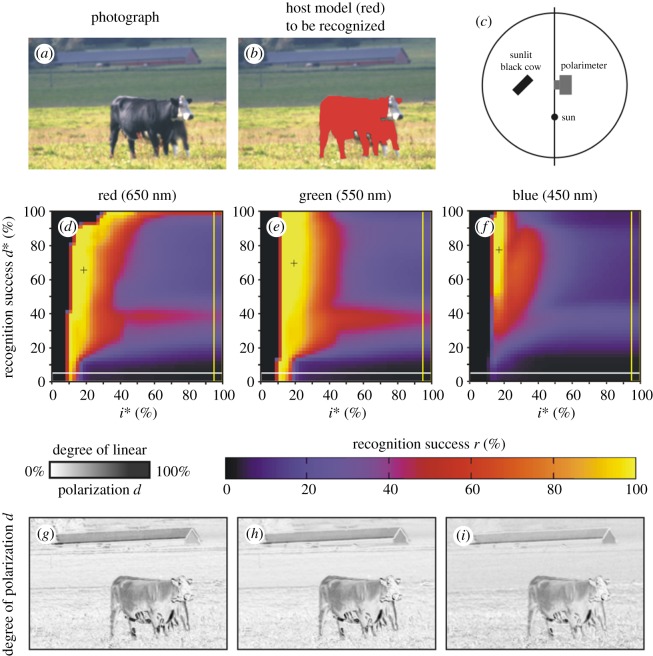
Scene 9. As figure 4 for a sunlit black cow. The arrangement is shown in panel (*c*) and the illumination conditions are given in table 1.

All the above findings are supported by the same results obtained for a living black cow in scenes 8 and 9 shown in figures 10 and 11, where she was shady and sunlit, respectively, with the same body position: (i) In any spectral range, the maximum of the recognition success *r* obtained with algorithm 2.3 for this cow was always much greater than that for algorithms 2.1 and 2.2 separated (table 2 and figure 2). (ii) In a given part of the spectrum, the shady cow (scene 8, figure 10*g*–*i*) polarized reflected light weaker than the sunlit cow (scene 9, figure 11*g*–*i*).

## 4. Discussion

In this work, we showed that polarization vision in horseflies is necessary for the detection of dark hosts: (vii) horseflies can distinguish dark-coated host animals from the dark patches of background vegetation by means of the degree of polarization *d*, because darker hosts reflect light with higher *d*-values than shady dark regions of bushes and trees (see the six functions listed in the Introduction).

Healthy host animals of horseflies (e.g. horses, cattle and other ungulates) have mainly a shiny, smooth coat with nearly parallel neighbouring hairs (apart from cases when, e.g. the hairs are long in order to ensure thermal isolation in winter, at times when horseflies do not occur) directed randomly (also partly to increase thermal isolation) resulting in a rough surface with a matt appearance. Shiny, smooth coats reflect light with higher degrees of polarization *d* than matt ones, because rough surfaces depolarize reflected light: the rougher a surface, the larger is its depolarizing (i.e. *d*-reducing) effect. According to the rule of Umow [39], the darker a surface in a given wavelength range, the higher is the *d* of reflected light. Therefore, black-coated host animals reflect light with the highest *d*-values. At a given reflector, *d* is maximal at the Brewster's angle. Thus, a polarization-sensitive flying horsefly perceives high degrees of polarization when it is looking at regions of the body surface of a dark-coated host animal from which light is reflected at and near the Brewster's angle.

In sunshine the greatest amount of sunlight is reflected from the specular direction, when the angles of incidence and reflection of sunlight are equal. From all other directions, the much weaker skylight is reflected. If the specular direction of reflection is the same or similar to the Brewster's angle, the degree of polarization *d* of specularly reflected sunlight is very high for dark coats. If a dark host animal is in shade, a given point of its body surface receives light from all possible directions from the environment (sky, vegetation and ground), the consequence of which is a low *d* of reflected light. These are the physical reasons for the fact that sunlit dark host animals reflect light with higher degrees of polarization than shady ones (figures 7*g*–*i*, 8*g*–*i*, 10*g*–*i* and 11*g*–*i*).

Contrary to the high *d*-values of light reflected from sunlit dark host animals, the degree of polarization perceived by an ommatidium of a flying horsefly looking at a shady dark region of bushes and trees is relatively low. A randomly oriented shady leaf blade receives light from all possible directions of its environment (sky and other leaves). The consequence of this is that there is no specular reflection, i.e. there is not any particular direction from which a leaf reflects light. Owing to this diffuse reflection, shady leaves of a dark region of vegetation reflect light with low *d*-values and random direction of polarization. An ommatidium of a horsefly eye averages the polarization of light received in its field of view. If the direction of polarization is not constant in this field of view (especially for scenes/targets being in a remote distance), the net averaged *d*-value is further decreased. Therefore, dark shady patches of vegetation reflect only weakly polarized light, the *d*-values of which are lower than those of light reflected from sunlit dark host animals of horseflies.

According to our experience (GH 2008--2017, unpublished data), horsefly females look for sunlit dark host animals, rather than shady dark ones or dark patches of the optical environment (mainly vegetation). Shady dark host animals and the dark patches of the environment are usually only weakly polarizing, while sunlit dark host animals are strongly polarizing, as demonstrated in this work. Thus, horseflies could find a sunlit dark host by means of the high degree of polarization of host-reflected light, distinguishing it from a weakly polarizing shady host and from dark environmental patches being inappropriate for blood sucking. Confirmation of this prediction by capturing horseflies with host models under various lighting conditions and background types could be an important task of future research.

As a first approximation, one may think that for horseflies the easiest approach would be to select host animals on the basis of the light intensity only, i.e. they could simply seek for dark patches of their environment. With this search strategy (algorithm 2.1), they would, however, also detect all dark patches of the background vegetation. This would be a non-efficient host-finding method. An alternative strategy could be that horseflies find hosts solely on the basis of the degree of polarization *d*, i.e. they could simply seek for highly polarizing patches of their environment. With this search strategy (algorithm 2.2), however, they could practically detect only hosts which are oriented in such a way that their body surface reflects light specularly resulting in high enough *d*-values. If the threshold d* were decreased, also many leaves, that are oriented appropriately for reflecting light with higher *d*-values, would be wrongly detected as hosts with this algorithm. Thus, both host recognition algorithms 2.1 and 2.2 are not ideal for efficient host detection, and would result in erroneous results and increased energy expenditure for the host-seeking behaviour. A more efficient solution to find hosts would therefore be expected to evolve in the horseflies.

We showed here that looking for sufficiently dark and polarized targets is a good strategy (algorithm 2.3) for horseflies to detect sunlit dark host animals, because the latter can effectively be distinguished from dark, shady and only weakly polarizing regions of the background vegetation. From our results presented in this work, we conclude that horseflies could use their polarization sensitivity to look for targets with higher degrees of polarization in their optical environment and thus to find more reliably sunlit dark host animals, instead of shady ones or instead of other dark environmental (e.g. vegetation) patches being distractive and inappropriate for blood sucking.

It is not known in which part of the spectrum horseflies sense polarization of light [1,32]. Therefore, we performed our present study in the red, green and blue spectral ranges. We found that the host recognition is most effective in the blue, because using the blue spectral range, horseflies can identify hosts in the widest range of intensity and degree of polarization. Although we could not perform imaging polarimetry in the ultraviolet spectral range, the reflection–polarization characteristics of our host model, black cow and the studied vegetations may be more or less similar to those measured in the blue part of the spectrum. Thus, we assume that our main conclusion, that degree of polarization helps horseflies to select host animals from the dark patches of the visual environment, is valid also in the UV.

Here we obtained experimental evidence that in a given part of the spectrum, the maximum of the host recognition success *r* depends on the alignment of the host model relative to the observer (horsefly, polarimeter) and the sun as well as on the illumination condition (sunny or shady) (table 2 and figures 2 and 3*j*–*l*, figures 4–11*d*–*f*). Thus, if the search algorithm 2.3 of a horsefly has fixed thresholds i* and d*, a host with an inappropriate alignment and illumination may not be recognized, even if it is sunlit and dark. This is, however, not a serious problem, because host-seeking horseflies fly randomly and they fly around targets that can be a possible host. During such a circumnavigation, there are several such directions of view from which a sunlit dark host reflects light with optical characteristics (*i* and *d*) that result in large enough host recognition success *r*, as shown here (figures 2–11 and table 2). Furthermore, it can also be envisaged that thresholds i* and d* in the visual system of horseflies can be adaptively changed according to the background and illumination conditions of the observed scene.

In this work, we demonstrated the importance of polarized light perception in the visual host location by horseflies. We would like to emphasize that other cues may also play an important role in host choice. For example, horseflies are lured by the movement of baits [11,40,41], thus their host animals are detected by motion too. Moreover, horseflies are very strongly attracted to hosts by odours [11,15–17,19,42,43]. However, Blahó *et al*. [44] showed that dark-bright stripes, for instance, disrupt odour attractiveness to horseflies, and thus the colour pattern of hosts has a dominance in the sensory system of host-seeking tabanids relative to host-specific odours (e.g. ammonia, CO_2_). Further studies are needed to determine the relative contributions of different cues in the host detection by different horsefly species.

Our conclusions presented here are based on imaging polarimetric measurements of different scenes and their usage in comparative evaluations based on various algorithms. In the future, these findings could be confirmed by entomological investigations, using, for example, the same host model in front of different vegetation patterns and comparing the number of tabanids attracted by this model among the different vegetation patterns.

## Data accessibility

Our paper has no supporting material. All data and research materials supporting our results are in our article.
